# What Women Want: Mental Health in the Context of Violence Against Women in Sri Lanka—A Qualitative Study of Priorities and Capacities for Care

**DOI:** 10.1177/10778012241230326

**Published:** 2024-02-23

**Authors:** Alexis Palfreyman, Kavitha Vijayaraj, Safiya Riyaz, Zahrah Rizwan, Sambasivamoorthy Sivayokan, T.H. Samanmalee Thenakoon, Madhubashinee Dayabandara, Raveen Hanwella, Delan Devakumar

**Affiliations:** 1 204274 Institute for Global Health, University College London, London, UK; 2 Independent, Colombo, Sri Lanka; 3 Psychiatry Department, 586186 Jaffna Teaching Hospital, Jaffna, Sri Lanka; 4 Department of Psychiatry, 63735 Faculty of Medicine, University of Colombo, Colombo, Sri Lanka

**Keywords:** violence against women, intimate partner violence, mental health, psychosocial support, Sri Lanka

## Abstract

Insufficient evidence guides mental health service development for survivors of violence against women in Sri Lanka. Provider and survivor perspectives on (1) what constitutes mental health, (2) quality of care, and (3) priority areas and stakeholders for intervention were identified through framework analysis of 53 in-depth interviews. Desired care is chiefly psychosocial—not psychological—prioritizing socioeconomic, parenting, and safe environment needs in non-clinical community settings. Our evidence points strongly to the need to strengthen non-mental health community-based providers as “first contacts” and reassessment of health system-centric interventions which neglect preferred community responses and more holistic approaches accounting for women's full circumstances.

## Introduction

An estimated 41% of women in South Asia are survivors (SVs) of intimate partner violence (IPV)—just one form of violence against women (VAW), which describes physical, sexual, emotional, and economic violence and/or coercive control by intimate partners, domestic, and non-partner perpetrators (Fonseka et al., 2022). Asia also has one of the highest burdens of modern slavery through which women face forced marriage, forced labor, and commercial sexual exploitation (Oosterhoff et al., 2018). In Sri Lanka, between 18% and 72% of women experience IPV, depending on background and IPV definition (DCS, 2020; WHO, 2018). Civil conflict, amid discriminatory gender norms, has also exposed a majority of women to abuse, trauma, and risk factors for poor mental health (Fonseka et al., 2022).

Mental disorders, as one manifestation of poor mental health, are associated with VAW (Oram et al., 2022). Meta-analyses show an increased risk of mental disorders associated with VAW including depression (OR: 2.77, 95% CI [1.96, 3.92]), anxiety disorders (OR: 4.08, [2.39, 6.97]), and post-traumatic stress disorder (PTSD; OR: 7.34, [4.50–11.98]; Trevillion et al., 2012). The VAW–mental disorder relationship is bidirectional, and both are also linked to previous abuse as a child (Oram et al., 2022). Global evidence also consistently demonstrates that childhood or adulthood exposure specifically to IPV heightens women's risk of suicidal ideation and attempts (Oram et al., 2022). In Sri Lanka, IPV creates a recognized vulnerability for developing depression, suicidal ideation, and/or behavior in perinatal women with intergenerational consequences (Palfreyman, 2019a, 2021), while VAW more broadly directly informs women's choices to use self-harm (Palfreyman, 2019b). Therefore, in addition to primary prevention of VAW, high-quality services for SVs are essential to support SVs’ mental wellbeing.

VAW services in Sri Lanka are variable in form and quality (TAF, 2016; WHO, 2018). Services are overwhelmingly (90%) delivered by local non-governmental organizations, 45% of which focus on gender equality and empowerment (Kodikara & Piyadasa, 2012). Geographically, services are mostly in urban centers, with uneven distribution and fewer serving directly post-conflict regions (Kodikara & Piyadasa, 2012). Around half offer counseling, though data on what this constitutes are absent, and formal mental health treatments are directed to the health system (Kodikara & Piyadasa, 2012). Sri Lanka's national health service aims to offer all residents mental health services, however, health expenditure is waning. Official mental health allocations remain unclear and entangled with other noncommunicable diseases (TAF, 2016). As little as 1.6% of the national budget is earmarked for mental health, primarily for psychiatric and curative services (Jenkins et al., 2012).

The need for SVs and providers to co-produce service development and assessment approaches responsive to SVs’ needs and priorities is increasingly emphasized in global best practices (Oram et al., 2022). Yet exploration of feasible, context-affirming service models and interventions must account for resource landscapes and the greater difficulty in accessing statutory mental health services in settings like Sri Lanka (Oram et al., 2022).

To inform the development of future quality mental health interventions tailored to SVs of VAW in Sri Lanka, we explored SV and provider views with two objectives to:

- better understand the mental health needs and preferences of SVs of VAW in two diverse locations in Sri Lanka and
- obtain suggestions for beneficial interventions, from diverse perspectives.

## Sri Lankan Context

Sri Lanka is a small island nation in the Indian Ocean. Its multiethnic and multireligious population of 21.7 million comprises chiefly Sinhalese (74.9%), Tamil (15.3%), and Muslim (9.3%) communities (DCS, 2012). Civil war (1983–2009) between the Sri Lankan State and the pro-secession group, Liberation Tigers of Tamil Eelam, introduced myriad social impacts including large-scale internal and out-migration and a redrawing of gender roles like rising female-headed households (26%; DCS, 2016). VAW is common, but difficult to redress through legal mechanisms due to the current policy landscape in which marital rape is not yet a criminal offense, girl-child marriage is sanctioned under plural family law and customary acts, and female genital cutting is practiced in certain communities (Fonseka et al., 2022; Ibrahim & Tegal, 2019; WHO, 2018). There are also claims of sexual violence perpetrated by combatants during the civil war (UNHRC, 2015).

## Method

### Study Setting

Two districts were selected for diversity in sociodemographics, migration patterns, conflict legacies, and availability of services. First, Jaffna District (population 626,000; Registrar General, 2021) faced protracted and centralized exposure to the civil conflict, with consequences for infrastructure and health, including high rates of girl-child marriage (31%) and IPV (>50%; Fonseka et al., 2022). Residents are mainly Sri Lankan Tamil (94%) and practice Hinduism (78.9%; DCS, 2012). Second, Kurunegala, a distal district during the war, has a predominately Sinhalese Buddhist (82.4%) population of 1.74 million (Fonseka et al., 2022; Registrar General, 2021). Kurunegala's labor emigration far exceeds Jaffna's, with implications for the mental health of female migrant households and those left behind (Knipe et al., 2019). Both districts have suicide rates above the national average, a potential indicator of a higher need for intervention; female suicide is particularly high in Jaffna (Knipe et al., 2017).

### Participant Recruitment

Diverse provider and SV perspectives were sought through four complementary strategies. First, study partners identified candidate district providers (individuals and organizations) through professional networks. Those not formally tasked with violence and/or mental health interventions were included as potential providers in a broader nexus of care. Second, we selected stakeholders representing (mental) health, medicolegal, special interest, and (non)government bodies from a situation analysis of existing VAW and mental health services previously conducted by our group. Third, participating providers connected interested service users (i.e., SVs) with the research team to discuss participation. Fourth, snowball sampling identified new participants, including non-service using SVs. One potential participant declined, while another did not attend the interview. Recruitment aimed for maximally inclusive perspectives. Inclusion criteria required participants to be 18 years plus; a current, former, or potential provider of mental health and/or violence support services, woman SV of violence, or both (i.e., dual provider-SV identity); resident of Jaffna or Kurunegala Districts; and demonstrating capacity to consent. In total, 53 participants contributed between October 2020 and April 2021.

### Research Team and Positionality

The all-women data collection team comprised a research lead (AP) and four primary data collectors (AH, ZR, KV, and SR), with experience in clinical medicine, social sciences, and development. All languages, ethnicities, religions, and relevant districts were represented, supporting sociocultural familiarity with a wide range of participants and our ability to meet their needs. Our study was guided by principles of critical feminist research particularly for mental health research with women in which attention to language, reflexive practice, representation of diverse voices, and mobilization of the evidence for social change were consistently revisited during and post research (Lafrance & Wigginton, 2019). We strove to promote inclusivity while aiming to minimize power imbalances throughout the study both with participants and each other.

### Data Collection

Data collectors completed study-specific training addressing research design, safeguarding, ethics, teamworking and wellbeing, and roleplay methods. Due to COVID-19, we remained responsive to participant preferences and government guidelines; earlier interviews were necessarily conducted via video- and voice call, while later interviews were safely held in-person as pandemic conditions improved, as in other pandemic-affected health projects (Vindrola-Padros et al., 2020). Interviews were held at convenient times and locations for participants, preserving privacy, limited interruption, and SV safety, particularly for remote scenarios. Data were collected by AH, KV, and SR in Jaffna and ZR and SR in Kurunegala. Interviews were audiorecorded, and conducted in participants’ preferred language and mode, given COVID-19. Each continued until information redundancy was achieved, typically in 1–2 h.

### Research Instruments

AP developed two complementary semistructured topic guides informed by global and local violence and mental health evidence (gaps) (Paphitis et al., 2022; TAF, 2016). Both explored local understandings of mental (ill)health and its relationship with VAW; and current and potential forms of mental health support including for SVs affected by conflict, migration, and disability. Tools were intentionally care-seeking (needs and preferences) orientated, minimizing requests for potentially re-traumatizing victimization narratives (Williamson et al., 2020). Unique to SVs and informed by (a) previous local evidence on coping strategies of women SVs of interpersonal and self-directed violence (Palfreyman, 2019b) and (b) Bruce et al.'s (1990) quality-of-care framework, locally illustrated cards aided conversations around support preferences including what impacts quality. Card Set 1 depicted 11 types of psychological, sociocultural, and healthcare resources employed by previous women research participants to ease mental distress, for example, economic and legal assistance. Set 2 visually presented Bruce et al.'s (1990) elements of quality care including offering choice, resource mix, information, provider competence, positive interpersonal dynamics, and care continuity. To our knowledge, this is the first application of Bruce et al.’s quality framework in violence or mental health research. Cards contained words and images to support low(er) literacy and were used as conversational prompts and priority-setting tools. All instruments were translated from English into Sinhala and Tamil and reviewed for accuracy of intended meaning by multiple team members fluent in both languages. AH and ZR piloted tools in Jaffna and Kurunegala respectively, with minor amendments to further demedicalize terminology. Finally, a “further support” service brochure was developed for participants. Trilingual tools were offered in physical and digital forms to meet participant preferences.

### Transcription and Translation

Interviews were transcribed verbatim by our research team; supplementary Tamil–English translation was provided by an additional female researcher (LS). All transcriptionists were trained in a uniform approach and subject to confidentiality agreements. Ten percent of all transcripts underwent quality assurance in which documents were swapped among language-matched team members to assess accuracy and completeness.

### Data Analysis

Framework analysis was selected for its flexibility for multidisciplinary teams with mixed qualitative experience (Parkinson et al., 2016). We followed five steps to organize, reduce, and identify relevant evidence for each objective (Parkinson et al., 2016). AP first randomly selected 10% of transcripts for manual familiarization (Step 1) to develop an initial thematic framework (Step 2). Transcripts and the working framework were uploaded to NVivo (QSR International, 2020) where ZR and KV separately tested a second random 10% sample. In collaboration with AP, they refined the framework, setting clear application guidelines until a priori and a posteriori codes consistently captured content across all sample transcripts. ZR and KV assigned the remaining transcripts’ text segments to appropriate categories (Step 3—indexing). AP, ZR, KV, and SR charted the data (Step 4)—moving it from transcript to matrix form and exploring across (participant-based) and down (category-based) the matrix, developing summaries for each (Parkinson et al., 2016). Finally, the research team interpreted the dataset (Step 5) by identifying categorical relationships and higher-level themes.

### Ethics

Ethical approval was granted by University College London (REF 2744/007), the Institute for Health Policy (Sri Lanka; IRB/2020-026), and the University of Colombo (EC-19-122). All participants received information sheets in their preferred language which data collectors orally reviewed. Participants signed separate consent forms; data collectors signed on behalf of consenting participants with low literacy and/or disability on three occasions. Identifiable data remain securely stored with the Institute for Health Policy; no identifiable data were transferred outside Sri Lanka and all transcripts were anonymized prior to secure storage. No incentives nor deception were deployed. Refreshments and compensatory transport allowance were provided. COVID protocols were observed for all in-person interviews. In-built safeguarding mechanisms were activated twice with preferred support provided to participants. External clinical supervision was available to the research team as needed, in line with best practice (Williamson et al., 2020).

## Results

Three themes organize participants’ needs and preferences for mental health support in the context of VAW: (1) understanding mental health and its relationship with violence, (2) quality of care, and (3) priority areas and stakeholders for intervention.

To better understand participants’ choices, we first describe their backgrounds including histories of violence and mental health. Illustrative quotations from service providers (SPs) and SVs aid transparency and interpretation.

### Participant Backgrounds and Histories of Violence

Thirty-two providers of all genders represented broad experience and service contexts (see Table 1); roughly a quarter had some training in mental health. Providers, being mostly women, could disclose a dual SV-provider identity, but chiefly contributed as providers. SVs aged from early 20s through 50s, and held varied marital, motherhood and socioeconomic statuses. SVs included but were not limited to war widows, heads of household, (former) housemaids, social activists, (mothers of) ex-combatants, day laborers, and—addressing shortcomings of previous VAW research—unmarried and transwomen, and those with histories of migration and disability (Oram et al., 2022; TAF, 2016).

**Table 1.** Participant Characteristics.

	Type of participant (*n*)	
Characteristic	Survivors	Service providers
**District**		
Jaffna	14	22
Kurunegala	7	10
**Gender**		
Woman	21	20
Man	0	11
Transgender	0	1
**Service setting accessed/provided**		
Community-based/nongovernmental organization	17	15
Independent therapist	1	1
Specialist service^a^	3	5
State sector—clinical	3	8
State sector—nonclinical	3	7
Traditional healer/charmer	0	1
Did not access any service setting above^b^	2	0
**Data collection mode**		
In-person	16	9
Video and/or voice call	5	23

*Note*. Service setting categories are not mutually exclusive and account for participants accessing/working in multiple settings.

a Supports subpopulations like women with disabilities, survivors of migration/labor exploitation, religious or ethnic groups, and transwomen.

b Survivors may have sought support through categories not included.

SVs were rarely impacted by VAW in just one setting or time point, with compounded exposure to “domestic violence, street … workplace harassment (and) … when accessing government services, sexual bribery … (in) sex work … a lot of violence (was) faced when reaching out to police, law, lawyers, at courts and all” (SP1, Kurunegala). Table 2 presents the many categories, interpersonal relationships, and settings in which VAW was encountered.

**Table 2.** Summary of VAW in Study Districts.

Type of violence	Perpetrator	Setting
Intimate partner violence	(Ex) husband	Household; social media and messaging platforms
Family violence (non-partner)	Mothers (-in-law)	Household
	Father	
	Siblings (-in-law, e.g., brother-in-law)	
	Uncle	
	Son	
	Other relatives	
Community and sexual violence and harassment	Employers, male and female colleagues	Workplace, for example, labor-intensive industries
	Male principals and teachers	Educational institutes, for example, schools, universities
	Male military personnel	Military checkpoints and bases
	Psychiatrists, nurses, counselors	Health services, for example, hospitals, clinics; local charities
	Government officers	Government institutions, for example, divisional secretariat
	Male community members	Street-based
	Male commuters	Public transport
	Police officers	Police stations
	Unspecified legal actors	Legal institutions, for example, courts
Cyber and tech-based violence	Social media users, (ex) intimate partners	Social media and messaging platforms, for example, Facebook and WhatsApp
Modern slavery/labor exploitation	Employers, recruitments agents	External destination, for example, domestic settings in the Middle East Internal destination, for example, daily wage settings

*Note.* VAW = violence against women.

IPV was reported as the most common form of VAW by 53% of participants, while family violence, involving non-intimate partners was cited by 38%. Intimate partners and strangers were reportedly increasingly deploying technology-aided violence:

(Her husband) is threatening her … he will post the picture on the internet and the woman is really suffering because she is panicked. So, these days, through electronic formats a lot of violence is happening. (SP3, Jaffna)

Labor exploitation by “unscrupulous” recruitment agencies pre- and post-migration and employer abuse during placements were significant challenges for migrant women domestically and internationally. Predominant forms of violence included physical (e.g., hitting), sexual (e.g., [marital] rape), emotional abuse (e.g., shouting, scolding, and accusations of infidelity), economic and emotional neglect; abandonment; and structural violence, for example, through state institutions: “… the courts made us stay together” (SV14, Jaffna). Perpetrators were chiefly men, particularly in households, with violence motivated by suspicions over women's (in)fidelity, maintaining “family respect” (SP21, Jaffna), and “if the girl doesn’t bring enough dowry, they will start to torture their wives” (SP17, Jaffna). Violence from family males was consistently associated with their alcohol and/or drug misuse.

### Theme 1: Local Understandings of Mental Health and Its Relationship With Being a SV of VAW

Providers mostly held a basic and medicalized understanding of mental health. Despite most lacking training in mental health, screening or diagnosis, they overwhelmingly described SVs as presenting with mental disorders like “depression,” “anxiety,” “PTSD,” and “somatization” (SP1, Jaffna) or that service users were “mentally ill/sick.” SVs, conversely, relayed mental health impacts of violence in feelings and emotional states like being “sad,” “unhappy,” “scared,” “angry,” or as “mental torture/pressure.”

Importantly, SVs also conceived of mental health as chiefly being about one's ability to perform functions with a “clear” mind and express oneself in “acceptable” ways, with poor mental health conferring the opposite. Functions included SVs’ ability to “look after” (SP4, Jaffna) and uphold honor and respect for their families, do jobs, problem-solve, and be resilient in the face of (financial) hardship, despite the violence. Functions like fulfilling one's (family's) basic needs and living without socioeconomic difficulties were core “for the mind to be at a good state to live”—a state poor(er) women SVs would not easily achieve: “if one has money, one can be happy” (SV5, Kurunegala). Positive mental health is manifested through emotions like being “happy” (SP1, Jaffna) and a physically “healthy body” (SP6, Jaffna). Expressions of poor mental health in others could include being sad or angry, crying, and abusing alcohol and other substances.

Participants uniformly perceived high mental health stigma, noting people with mental health difficulties were viewed as “abnormal” (SP1, Jaffna). Notably, a gendered distinction was made about public perceptions of mental health difficulties, where men's were due to external factors like the “stress” of occupying the breadwinner role, while women's were typically attributed to internal flaws including possession:

… if he behaves like that (expressing mental suffering), they think it's because … he is earning and stressed out. When it comes to women, people think … something has gotten into them (spirit possession). (SP19, Jaffna)

With SVs’ backgrounds in mind, we turn to what women want to support their mental health. Priorities for care were not just about resources, but relationships, with preferences for six areas of assistance, and particular stakeholders and modes of engagement. Participants expressed that, ideally, responses should work in service of three outcomes for SVs: (1) fulfillment of basic and safety needs which would reduce material and psychological suffering, (2) empowering SVs to disclose their problems freely, supporting help-seeking, and (3) equal opportunities and recognition for women which would foster “a good mental health state, (and) them contributing to (community) development” (SP6, Kurunegala).

### Theme 2: Achieving Quality of Care in Practice

Through priority-setting discussions using visual prompts presenting Bruce et al.'s (1990) fundamental elements of quality of care, SVs conveyed perceptions of quality and how quality priorities may function in action to encourage initial and ongoing engagement with available providers and for improved outcomes. Their perspectives may contextualize subsequent preferences for interventions and stakeholders. Rather than viewing the quality elements of (a) choice and (b) appropriate resource mix, (c) quality information, (d) provider competence, (e) positive provider–client relations, and (f) continuity of care as independent, participants described a recipe for quality where provider attitudes, choice and resource mix, and competence were interdependent *prerequisites* to achieve their top priority of healthy provider–client relations, laying the groundwork for subsequent progress in healing.

SVs chiefly wanted providers who were respectful, non-judgmental, trustworthy, and “kindly” even if solutions or other resources were not immediately available: “I thought if I go there, I will immediately get some relief. Not anything else. Not even money” (SV5, Kurunegala). Beyond the material and monetary resource mix, choice also meant being flexible and accommodating, as SVs could not always anticipate nor mitigate competing pressures and setbacks. This was especially important for women with disabilities, trans, and ethnic minority SVs. Critically, SVs consistently centered *emotional* intelligence, empathy, and a provider's ability to deconstruct problems as core provider competence often over technical capacities, for example, knowledge, qualifications, or experience. Combining these three elements produced the best dynamics for (ongoing) engagement:

I just wanted someone who knew what they were doing … I had (a) lot of different strands that contributed to my mental health. So someone who could unpick the strands without messing it up even further … somebody who would actually be patient … non-judgmental. (SV4, Jaffna)

With this healthy interpersonal foundation, SVs were (more) ready to build upon early engagement, seeking or returning to services that could provide (new) quality information and support with “how to solve a problem and how to take correct decisions during the problems” (SV3, Jaffna). Thereafter growth was encouraged by helping women expand to more sources of support and sometimes identifying a mutual approach to continuity of care without premature transition toward independent management: “We keep in touch … they connect me to other help … He has suggested weaning off … but I’m still not there yet so I prefer meeting once a week” (SV4, Jaffna). Participants all desired providers to attend to “deeper personal issues,” not just “putting a plaster” (SP2, Kurunegala), acknowledging this requires providers commit to quality.

### Theme 3: Priority Areas for Intervention

Six priority areas for intervention and resource provision were identified for improved psychosocial and mental health outcomes including economic, legal, parenting, health, community, and safe environment assistance.

#### Economic and Material Support

Women's ability to make meaningful changes, including “walk(ing) out of the problem (abuse)” (SV1, Jaffna), is heavily shaped by financial (in)security and (in)dependence. Sustained economic support could take multiple forms. First, equipping women with new skills and knowledge may support “how … you start a career … your business,” “investment planning,” and “marketing” skills or products (SP13, Jaffna). Second, providers could identify economic opportunities suited to SVs’ circumstances, like appropriate job training and networks offering economic and social protection. For example, women in one service were supported to join a collective exporting locally made products, generating income, while also reducing women's exposure to “harassment … when it is as a group” (SP13, Jaffna).

In other instances, highly targeted material support from government or nonprofit services could yield personally meaningful impacts for individuals. One SV with a disability felt “if (she) had a motorbike, it will be helpful” (SV8, Jaffna) for physical independence, ability to work, earn and access resources, and her mental wellbeing.

#### Support With Children's Welfare

Intimately linked to economic needs, and reflecting women's critical and disproportionate role as primary carers, participants underscored a need for assistance with children. Many emphasized a sense of responsibility for their children's future outcomes, despite limited hopes for themselves: “(I) want to educate my child. My life is over, but when it comes to my daughter's life, I am still not sure, it is still a question mark” (SV6, Jaffna). Support to identify (educational) resources to “provide for children (including) a safe environment to live in … to be able to educate … are the things (women) need” (SV5, Kurunegala), facilitating upward mobility, especially for those living in poverty.

#### Legal Support

Legal services were consistently viewed as the least attractive and “money-consuming, time-wasting” (SP9, Jaffna) mechanism for support. A high-level, SV-centered systemic transformation was judged necessary to mete out justice for SVs including legal reform and effective implementation of existing VAW-relevant laws: “Punishments and laws should be made stricter” (SP3, Jaffna).

Three key needs were isolated. First, providers could directly address instances where “victims are victimized again and again and not the perpetrators” as a normalized feature of legal processes, with a longer-term goal to “develop a new model … practiced on the ground” (SP3, Jaffna). Second, organizations’ ability to offer accompaniment services or clearly “show (SVs) the way … structures … what benefits (they) will receive” (SP9, Jaffna), would help SVs navigate complex processes and spaces, including and especially the police. Third, efficient administration was desired including timely connections to key legal (human) resources, and convenient clerical processes like “reduc(ing) the paperwork” and other steps “postponing or neglecting” (SP6, Jaffna) case progression. All three require providers possess functional literacy in the country's laws, legal processes, and stakeholders to ensure swift and appropriate referral as a *minimum* service, but demand coordinated and sustained advocacy as a block to realize truly systemic improvements.

#### Health Support

Health support needs were discussed as (a) practical improvements and (b) specialist services to routinely include in SVs’ care. Practically, participants lamented a deficiency in Tamil-speaking providers, regionally and within services in mixed areas, with “clinical psychologists in Tamil medium” especially lacking (SP19, Jaffna). Participants desire long-term investments to scale trilingual services, including a “cadre of counselors,” but better knowledge of other Tamil-speaking providers from diverse services could improve referral processes short-term. Participants also highlighted the need to educate providers, particularly clinicians, on the multiple ways (sequelae of) VAW presents within the health system, as women “indirectly present … where the major factor is … violence”: “The doctor … said … this lady is … going home and again she is coming … I anyways knew … after getting hurt, she gets admitted … this is how her coping mechanisms works” (SP3, Jaffna). Health workers’ inability to identify links is currently resulting in missed opportunities to engage SVs in vulnerable moments.

Three specialist services were viewed essential for supporting comprehensive health and healing for SVs. First, sexual and reproductive care was underscored by women, which may include access to services (e.g., screening), products (e.g., contraception), or being offered information because “sometimes women who face abuse … don’t talk about it due to shame” (SV1, Kurunegala). Second, access to counseling for women, and third, effective substance abuse treatment for male partners, “teenagers and school children … getting addicted” are necessary to support wellbeing of the whole family. Amid concerns of “increasing rates of alcoholism and violence by men” (SP11, Jaffna), providers worried that without deaddiction support, “our future community will get ruined” (SP6, Jaffna).

#### Community Support

Robust, multipronged community-level support for SVs was a central aspiration by all for improved care and outcomes. Participants overwhelmingly spoke in terms of what *should* happen, rather than what is happening and should continue. This subsection is then chiefly about unmet objectives for prevention and intervention for VAW and mental health, as “when everyone in society works together in unity only there will be success” (SP9, Jaffna).

Participants voiced a clear aligned preference to give and receive support in community-based settings, over and above even targeted goals for developing clinical mental health services for SVs. Myriad (potential) benefits of investing in community members to formally respond to both violence and mental health were noted. First, some providers felt local volunteers and community members, who could be trained in psychosocial interventions, would be more aware of community and personal circumstances, have greater interest in SVs, and be better positioned to “talk to someone continuously” than more removed clinical services: “They will … understand these people's situation, according to that they will go to do the interventions” (SP13, Jaffna). Further, participants conferred that women are more comfortable within their own community spaces which may foster better quality of care through stronger provider–client foundations, though perceptions were mixed as to whether confidentiality would be upheld and community providers “might gossip about me to someone else,” creating “trouble” (SV4, Jaffna). Community-based providers may be more flexible than some clinical mental health services, better able to accommodate SVs’ schedules and locations, perhaps through offering home visits and care outside typical working hours which “other (clinical) institutions don’t have” (SP13, Jaffna). From a practical perspective, and tied with economic support, trained community-based services should support a comprehensive approach to identifying and accessing relevant resources, including state benefits like social welfare payments, loans, and more: “They will guide … you can go and ask for a loan … Samurdhi (benefits) … You can raise a goat? Like this they will say” (SP13, Jaffna).

Community actors were also viewed critical for mobilizing SVs and those around them to strengthen proximal social networks and interpersonal support. Facilitating development of local SV networks could be beneficial for women to seek peer-to-peer support with livelihoods, mental health, physical companionship, and swapping “skills this one doesn’t have”: “As a group … they will be a comfort to one another … in a physical way also help will be there … psychologically for them to solve their own problems, it will be an opportunity. A third party won’t be needed” (SP13, Jaffna). Community leaders could build capacities in mental health to diffuse messages around resources. Working with families to “change a lot with the parents’ mindsets and their parenting method” (SP6, Jaffna) toward adult child SVs, particularly those who have left abusive partners and may be living alone, was stressed for SV recovery. Finally, several SPs shared a preference for involving “those who are in the decision-making position, both men and women leaders” and male perpetrators in interventions, as “until we work with those who cause the problem, we cannot solve the problem” (SP19, Jaffna). This could identify root causes for violence and minimize opportunities for re-offending:

A girl was abused by a male teacher … but … what the authorities do is they just transfer the person …so this is a very stupid thing. So here we are giving a chance for another population to get affected by the same person. (SP3, Jaffna)

Ultimately, communities should also play a central role in first preventing violence and its mental health sequelae. Many participants underscored the importance of recognizing the interconnected nature of violence with “living in a culturally bound society … (which) won’t accept (women's) decisions,” and systems within which “people are not really understanding that (certain forms of abuse) is a violence” (SP3, Jaffna), prohibiting help-seeking. Norm-changing initiatives “from the school level” were widely called for to facilitate a greater valuing of women and girls, to recognize and name all forms of violence, and transform ideologies that dictate “women bear with all this and live” and that “problems of the house shouldn’t be told outside” (SP7, Kurunegala). Continuous empowerment from girlhood “to develop young women leaders … (with) a strong voice for themselves” and move into “decision-making positions” (SP19, Jaffna) was a long-term community goal, including at organizational levels. Some participants encouraged capacity strengthening initiatives teaching self-awareness of strengths and capabilities and expanding women's social networks for greater independence: “Instead of providing fish to someone who is in hunger, giving them a fishing net would be more valuable” (SP3, Kurunegala).

Such desired cultural transformations could conceivably be achieved through education initiatives in communities, diffused through different mechanisms including newly married couples, families, schools, and health actors. Starting at home and in the “education system (which) needs to develop a lot” (SP3, Jaffna), participants aspire to see girls supported by families and educators to pursue formal higher education and, later, paid employment over early marriage, as “if girls get married before having a job … (they) will only have some few friends … all girls” (SV1, Jaffna) which may leave them more vulnerable to VAW and other social challenges. Knowledge on identifying violence, national relevant VAW laws, and “awareness on sex and marriages … should be taught in school at different grades” (SP3, Jaffna). Providers also suggest core mental wellbeing curriculum at A-level and university, though that may miss a substantial proportion of young learners. Beyond the school arena, “psychosocial education (from) … existing structures of service providing people must get together and do this” (SP9, Jaffna) in community circles to support those beyond schooling age. Borrowing from faith-based premarital counseling models, married couples could be supported to access pre-parenthood counseling: “We need to change a lot with the parents’ mindset and their parenting methods. In Catholic religion … they have special trainings to attend. So we need to introduce such kind of things in our community as well” (SP6, Jaffna). Mainstream and social media, including “newspapers and television programs,” should be leveraged to carry out “a large awareness program,” in which “the Ministry of Health (MOH) needs to be involved” (SP7, Kurunegala) to spotlight different service options and access points. Finally, stigma-busting initiatives to tackle “very demeaning” and stereotypically sexist treatment of subgroups like (war) widows and transwomen must be pursued to “serve (their) situation” and inform communities of their rights and support needs:

Even friends at my office, when they speak of (war) widows … they say these women get a huge compensation without using that wisely, they’re being adulterous, spending unnecessarily and so on … we all need to positively look at it and support them. (SP2, Kurunegala)

#### Operating in Safe Environments

Cross-cutting and synergistic with improvements in previous areas is the need “to provide … a safe environment to live in” (SV5, Kurunegala). Safety can be a nebulous and deeply personal concept. In our study, safety was desired both in terms of access to “safe spaces” and safety planning to gain distance in violent moments and/or relationships. Safe spaces could include “daycare facilities … at the rural small villages … through community centers they can establish” to offset a lack of safe childcare options: “There is no safe place to put their children” while women work (SP4, Jaffna). They were also defined as settings in which SVs could speak freely and be protected from further violence, with a “client-centered approach so … (a) woman will always feel that she is kept safe” (SP12, Jaffna). Provider safety planning requires greater interagency transparency to know onward services are “conducted properly” and free of violence themselves: “If we want to send a lady to a safe house, we have to consider a lot of things … like any … violence there” (SP15, Jaffna). Finally, participants want more efficient and safer “independent transport” (SP8, Jaffna) to and from services and home visits, including for women with disabilities.

#### Preferred Stakeholders and Modes of Delivery

Providers and SVs lastly reflected upon stakeholder suitability and trustworthiness to offer care in these six priority areas and how they might best deliver it. First, “grassroots women's organizations led by women” (SP1, Jaffna) were deemed most knowledgeable and appropriate to champion service provision, yet a “women-only” approach was dissuaded as “male leaders” were viewed critical for shifting male perspectives and “work(ing) together with the perpetrators” (SP19, Jaffna). Next, greater development and direct involvement of “survivor-led organizations (with) … expertise and experience” (SP11, Jaffna) was preferable to minimize testimonial injustice toward SVs in mental health and psychosocial care. Third, scale, distribution, and targeting of resources by region was perceived essential, especially for historically underserved populations like conflict-displaced women “who have faced difficulties for a long time” (SP9, Jaffna). Key services at the division level may encourage help-seeking given improved geographic proximity. Finally, multimodal service delivery, using in-person and “new methods and technologies” (SP6, Jaffna) may support women with disabilities and transport limitations, while being conscious not to “fail some women” (SP4, Jaffna) by shifting too far toward remote mediums.

## Discussion

Our work sought to identify how women SVs and providers of violence and mental health assistance understand SVs’ mental health needs, and their preferences and suggestions for interventions in Sri Lanka. We selected two distinct districts, serving diverse ethnic, socioeconomic, and conflicted-affected populations, exploring VAW more comprehensively and beyond the dominant research focus on IPV or domestic violence (Oram et al., 2022). Our findings confirm that VAW occurs within an interconnected nexus of social stressors concerning economic insecurity and dependence, insufficient legal and health support, concerns about parenting, stigma, and unsafe environments for women SVs (Huq et al., 2021; Moulding et al., 2020; Oram et al., 2022). They also underscore women's exposure to violence in Sri Lanka is multipronged and cumulative, emanating from multiple sources, spaces, and over time. Participants expressed a need for more comprehensive support with a preference for community-based, integrated care, offering SVs mental health and economic, legal, and childcare assistance. Both providers and SVs consistently emphasized the role of informal support, which may reflect low confidence in formal services (Eastern Social Development Foundation, 2018) and/or dissatisfaction with narrow biomedical mental health interventions (Oram et al., 2022). We reflect on the importance of local and gendered understandings of mental health, and implications for services and interventions.

### Understanding Mental Health Needs—A Gendered Credibility Gap

SVs widely conceptualize mental health as their ability to perform often gendered functions with regard to their jobs and fulfilling needs of their children and family, while identifying poverty as an exacerbating risk factor for mental health difficulties post-violence. SVs situated mental health and wellbeing largely in terms of social determinants and personal contexts. Despite prior assertions that such framings indicate poor mental health literacy (Gaiha et al., 2020), SVs’ conceptualization aligns with definitions like World Health Organization's (2022), which recognizes the ability to “work productively” and “contribute to community” as vital components of good mental wellbeing. Women's wellbeing is embedded and existing within a complex context, further compounded by factors such as disability, conflict, and migration (Oram et al., 2022).

SVs’ sense-making of mental health is a partial reveal of the persistence of the “credibility gap”, that is, the common divide in focus, language, and ultimately prioritized interventions between mental health service users and (usually clinical) SPs (Patel, 2014). In our study, SVs identified multiple needs of daily living, going beyond or instead of diagnosis and treatment for mental disorders. SPs, including those with non-clinical backgrounds, contributed further evidence for a credibility gap as they more often associated mental health with psychological/psychiatric diagnoses and symptoms with less emphasis on women's realities and contexts. Clinical providers were perceived to hold a narrower conceptualization of mental health, emphasizing intrapsychic experiences and functioning, reflecting local practice wherein mental health is primarily managed through medical interventions (Fernando et al., 2017). A biomedical approach to understanding and addressing mental health challenges in SVs of VAW is increasingly challenged, as the current model may individualize and decontextualize the causes, and consequently appropriate treatment of mental health difficulties (Moulding et al., 2020; Oram et al., 2022). This then detaches women's distress from the violence and control that caused it and pathologizes women and their oppression, placing the locus of responsibility for management and recovery on them (Oram et al., 2022). Women's priorities did not reflect stereotypical “mental health” interventions, underscoring a more inclusive desire to relieve *social* suffering as a way of supporting their mental wellbeing. There is consequently a need to expand awareness and appreciation of less “narrowly defined biomedical constructs and treatments” (Patel, 2014, p. 16) among providers, including and perhaps especially clinicians, to one which better accounts for women's priorities of support with daily living and interpersonal functioning.

Conversely, an overly relational view of mental health is not without its potential shortcomings. Our finding that women strongly associate their mental wellbeing (sometimes *only*) in relation to those around them suggests women may be discounting their own individual experiences as invalid and/or insignificant against interpersonal needs. Women tethering their mental wellbeing to the ability to express themselves in “acceptable” ways, further reveals the way cultural and gendered norms impact their mental health, safety, and help-seeking behavior (Amarasinghe & Agampodi, 2022; Palfreyman, 2019a, 2019b; Silva et al., 2022). For example, recent evidence on mental health in the perinatal period in Sri Lanka shows that while women would raise concerns with husbands and in-laws, they expect to be met with scolding or minimization of their distress (Amarasinghe & Agampodi, 2022). This expressed expectation for their individual suffering to be invalidated likely impacts women's choices to disclose at all.

Both the overly relational and individualistic models of mental health and its care are ultimately shaped by strong gendered perceptions of how women “should” be or not in the context of violence and mental health (Oram et al., 2022). Previous local research has raised concerns that SVs’ perceived deservedness to receive support for their mental health, including suicidal ideation and/or behavior, is often influenced by providers’ assessments of whether and how women perform their womanhood—and suffering—in culturally acceptable ways (Palfreyman, 2019a). Both models distinctively penalize women in mental health discourses as either failing in a relational role or as internally broken. Current interventions and responses by providers in this study were noted to largely overlook women's circumstances and reactions in the broader context of gender inequalities that underpin and fuel violence. Addressing social and cultural assumptions and standards for gender, violence, and trauma responses, which foreground women's understanding of mental health and their priorities, is essential for delivering SV-centered care and dismantling wider social structures creating SVs in the first place (Oram et al., 2022).

### Service Improvement

In line with emerging evidence from Sri Lanka (Eastern Social Development Foundation, 2018), our findings foreground an undeniable gap between SVs’ priorities and the services currently being offered. This is linked to both the range of services provided and where they are situated.

#### Where and How?

Presently, the model of care delivered to SVs of VAW through the state health sector predominantly involves one-stop centers (i.e., Mithuru Piyasa/Natpu Nilayam). Non-governmental organizations also provide vital, out-of-hospital services despite not receiving state program or policy support (Guruge et al., 2015). Sri Lanka's centralized health system response to VAW is commendable and has made considerable progress compared to its regional counterparts (Sikder et al., 2021), but must be understood in the context of an already overburdened health system, facing a shortage of specialist mental health professionals to meet population needs (TAF, 2016). This has been exacerbated by the pandemic and evolving socioeconomic crises impacting the country. Despite a concerted push to decentralize mental healthcare provision, services are still predominantly concentrated within clinical institutions (Fernando et al., 2017). Meeting multiple SPs across multiple access points can result in women ceasing to access services altogether (Eastern Social Development Foundation, 2018), emphasizing the need for more efficient, multifaceted care in one place. A systematic review of LMICs has, however, highlighted several barriers in current one-stop center models (Olson et al., 2020), including insufficient resources, inadequate standardized operating procedures and monitoring, poor quality of care, and multisectoral collaboration. Placing the service within a clinical setting may also risk perpetuating the message that service users are “mentally sick/ill,” a known barrier to women disclosing distress and accessing support (Silva et al., 2022). Concerns about the quality of both integrated and standalone local services have been documented (Eastern Social Development Foundation, 2018; Wijegunasekara & Wijesinghe, 2020). Our findings indicate the problem could lie with the targeted, vertical nature of the current approach to women SVs, which misaligns with both women's manifold priorities for care and their perception that care quality is dependent upon healthy provider–client relationships achieved through a synergistic process. Mental health interventions that offer medication and counseling in silo then fall short of facilitating long-term thrivability among SVs (Moulding et al., 2020), borne out of and perpetuating the “credibility gap” between SVs and current psychosocial SPs (Patel, 2014). Quality care is also not viewed by SVs as a formulaic or linear process of inputs like an assembly line, but a dynamic relationship, wherein providers must possess capacities to navigate parallel action and setbacks, and assess SVs’ readiness for interventions and resources at different time points. Vertical and separate interventions do not lend themselves to this sort of relationship development. Our results therefore indicate a need for community-based, one-stop care centers, that offer a wide range of care options for women SVs in one place with agile, emotionally competent providers.

#### What?

Participants identified multiple services and six priority areas of intervention to best support improvements in SVs’ mental health. These ranged from economic to legal interventions and welfare services for their children. There is a need to deliver interventions targeting multiple areas in a holistic and organized way, and such integrated, multifaceted models have been tested in the context of sexual and gender-based violence in other LMICs with significant promise (Bennett et al., 2017). Interventions aimed at increasing SVs’ mental wellbeing through economic interventions like cash transfers, microenterprise assistance, and financial empowerment have shown effectiveness (Spangaro et al., 2021; Zimmerman et al., 2021) while integrating legal support to counseling and crisis intervention has also demonstrated potential in socioculturally similar India (Daruwalla et al., 2015). Integrated health interventions such as violence and sexually transmitted infection screening, contraception, community-delivered mental health support, and psychological therapies have produced benefits like increased identification of VAW, reduced re-exposure to some types of VAW and improved health in SVs (Lewis et al., 2022). SV safety strategies are recommended through the development of a secure therapeutic alliance and efforts to equip and empower them with skills to navigate their situations, in addition to safety planning interventions and establishment of safe spaces (Paphitis et al., 2022; Stern & Carlson, 2019). Evidence for parenting and child welfare support remains scarce in the context of VAW, though interventions supporting mother–child dynamics and motherhood as part of some women's identities are desired globally and by SVs in this study (Paphitis et al., 2022). Broader social and community interventions aimed at empowerment, transforming gender and behavioral norms, and addressing alcohol use among perpetrators have delivered some reductions in VAW in Sri Lanka and other LMICs (Bourey, 2015; Herath et al., 2018; Kirk et al., 2017). Targeting reductions in VAW and other recognized social drivers of depression and self-harm among Sri Lankan women, like male partner substance misuse, may both prevent and alleviate existing distress among SVs (Palfreyman, 2021).

Barriers exist to implementing wide-ranging services that address social determinants of women's mental distress, not least limited resources within the national health service (TAF, 2016). As cultural beliefs and values may become embodied institutionally through a bidirectional relationship, models of care and interventions should holistically tackle economic, political, and social processes that perpetuate VAW, in order to provide women with long-term solutions and support (Arroyo et al., 2016). It is imperative that attention and by extension funding and resources are directed toward addressing the structural causes of poor mental health and distress, in parallel with investment in one-stop centers (Chapman et al., 2020).

### Strengths and Limitations

Our study contributes a rare and much-needed analysis of comprehensive mental health needs among SVs of different forms of VAW in Sri Lanka. Obtaining views of both SVs and providers also offers diverse perspectives on how services could be set up and identifies clear problems in current service forms and functions. Privileging two distinct districts, with their own language, culture, and conflict histories, expands evidence on needs and possibilities for mental health care beyond the common focus on Sri Lanka's urban and better-resourced centers.

There were, however, limitations. Mixing data collection mode was necessitated by COVID-19 as in other recent global health research (Vindrola-Padros et al., 2020). While no participants declined to take part citing lack of technology access, mixed modes, and particularly remote data collection may have introduced reporting and sampling bias. Women with disabilities and transwomen were few in our sample, as were faith and traditional healers, limiting our interpretations of their respective experiences and potential contributions to reimagining mental health care in the context of VAW. We encourage future research which intentionally expands the involvement of these groups.
